# The Development of an Infrastructure to Facilitate the Use of Whole Genome Sequencing for Population Health

**DOI:** 10.3390/jpm12111867

**Published:** 2022-11-08

**Authors:** Nephi A. Walton, Brent Hafen, Sara Graceffo, Nykole Sutherland, Melanie Emmerson, Rachel Palmquist, Christine M. Formea, Maricel Purcell, Bret Heale, Matthew A. Brown, Christopher J. Danford, Sumathi I. Rachamadugu, Thomas N. Person, Katherine A. Shortt, G. Bryce Christensen, Jared M. Evans, Sharanya Raghunath, Christopher P. Johnson, Stacey Knight, Viet T. Le, Jeffrey L. Anderson, Margaret Van Meter, Teresa Reading, Derrick S. Haslem, Ivy C. Hansen, Betsey Batcher, Tyler Barker, Travis J. Sheffield, Bhaskara Yandava, David P. Taylor, Pallavi Ranade-Kharkar, Christopher C. Giauque, Kenneth R. Eyring, Jesse W. Breinholt, Mickey R. Miller, Payton R. Carter, Jason L. Gillman, Andrew W. Gunn, Kirk U. Knowlton, Joshua L. Bonkowsky, Kari Stefansson, Lincoln D. Nadauld, Howard L. McLeod

**Affiliations:** 1 Intermountain Precision Genomics, Intermountain Healthcare, Salt Lake City, UT 84107, USA; 2 Department of Pediatrics, University of Utah, Salt Lake City, UT 84108, USA; 3 Center for Personalized Medicine, Primary Children’s Hospital, Intermountain Healthcare, Salt Lake City, UT 84113, USA; 4 Department of Pharmacy, Intermountain Healthcare, Salt Lake City, UT 84107, USA; 5 Humanized Health Consulting, Salt Lake City, UT 84102, USA; 6 Brainspin, Salt Lake City, UT 84123, USA; 7 Transplant Services, Intermountain Healthcare, Salt Lake City, UT 84107, USA; 8 Department of Bioinformatics and Genomics, Pennsylvania State University, University Park, PA 16802, USA; 9 John Hopkins Genomics—DNA Diagnostics Laboratory, Department of Genetic Medicine, John Hopkins University School of Medicine, Baltimore, MD 21205, USA; 10 Ambry Genetics, Aliso Viejo, CA 92656, USA; 11 Department of Cardiology, Intermountain Healthcare, Salt Lake City, UT 84107, USA; 12 Department of Medical Oncology, Intermountain Healthcare, Salt Lake City, UT 84107, USA; 13 Department of Surgery, Intermountain Healthcare, Salt Lake City, UT 84107, USA; 14 School of Medicine, University of Utah, Salt Lake City, UT 84132, USA; 15 Department of Endocrinology, Intermountain Healthcare, Salt Lake City, UT 84107, USA; 16 Digital Technology Services, Intermountain Healthcare, Salt Lake City, UT 84130, USA; 17 DeCode Genetics/AmGen, Inc., IS-101 Reykjavik, Iceland

**Keywords:** precision medicine, population health genomics, genome sequencing, population sequencing, healthcare infrastructure, genomic medicine

## Abstract

The clinical use of genomic analysis has expanded rapidly resulting in an increased availability and utility of genomic information in clinical care. We have developed an infrastructure utilizing informatics tools and clinical processes to facilitate the use of whole genome sequencing data for population health management across the healthcare system. Our resulting framework scaled well to multiple clinical domains in both pediatric and adult care, although there were domain specific challenges that arose. Our infrastructure was complementary to existing clinical processes and well-received by care providers and patients. Informatics solutions were critical to the successful deployment and scaling of this program. Implementation of genomics at the scale of population health utilizes complicated technologies and processes that for many health systems are not supported by current information systems or in existing clinical workflows. To scale such a system requires a substantial clinical framework backed by informatics tools to facilitate the flow and management of data. Our work represents an early model that has been successful in scaling to 29 different genes with associated genetic conditions in four clinical domains. Work is ongoing to optimize informatics tools; and to identify best practices for translation to smaller healthcare systems.

## 1. Introduction

The use of genomic analysis has expanded dramatically over the past decade, with over 3500 genes associated with disease risk, diagnostic determination, and therapy selection. This has seen application in oncology, behavioral health, cardiology, neurology, medication safety, the neonatal intensive care unit and many areas of pediatrics. Multiple research efforts are underway to expand the implementation of genomic sequencing to newborn screening as well as for other areas of health screening [1,2,3,4,5], suggesting that the genome sequence will become part of the standard medical record. Rapid advances in next-generation sequencing have made the use of genomic testing a promising option for population health. The price of Whole Genome Sequencing (WGS) has been dropping precipitously and is now available for less than $500, well below the once sought-after benchmark of the “$1000 genome” [6]. The decrease in price has led to increased clinical adoption of WGS and increased understanding of the variants detected from WGS. With the technological advances in sequencing, several large studies have begun work on the clinical use of whole genome and exome sequencing, including Geisinger Health and Population England [7,8]. However, one limitation in the use of WGS for population-based health is that the sequencing, and the clinical care, are often occurring in separate settings.

In 2019 Intermountain Precision Genomics (IPG), part of Intermountain Healthcare (IH), launched the HerediGene initiative, including a partnership with DeCODE Genetics and Amgen Pharmaceuticals to enroll up to 500,000 patients to assess associations between WGS results and population health endpoints [9]. IH provides healthcare in 33 different hospitals and almost 400 clinics across 9 states in the Intermountain West. With HerediGene there was a unique opportunity to test the real-world use of WGS in population health, identifying serious areas of disease risk in often healthy participants. This effort has served as the development ground for IH’s population genomics framework, needed to support the manageable integration of genomic data into the healthcare system. Through the HerediGene Return of Results Program (HGROR) the patient’s sequence information is used clinically to inform patient management and prevent disease. In our first 26,302 participants with WGS, we have demonstrated tremendous potential to impact patient care. The need for a population genomics framework is compounded at IH by other clinical genomic initiatives. With the increased uptake of genome and exome sequencing, in addition to increased diagnostic yield, there are a significant number of secondary findings that are being returned to patients.

Medically actionable secondary findings have been recognized as a major barrier to scaling clinical WGS because of the resources required to manage these findings [10]. Having a population genomics framework within the healthcare system to manage these findings is a vital solution to this problem and a successful framework reduces the cognitive load, effort, and stress for a testing provider. Significantly, a population genomics framework within the healthcare system can assure more consistent and complete care for patients with reportable findings. Through our genomic initiatives at IH, we have encountered the significant need to develop an infrastructure to manage these patients firsthand. Healthcare systems and electronic medical record systems as they currently exist are not designed to manage these types of data or workflows. Scaling the processes required to manage care related to these patients’ genomic findings is not currently supported by any commercial vendors’ software that we are aware of. To meet our internal informatics need, we have developed a population health genomics program, designed workflows, and implemented custom-built informatics solutions to manage these patients and their clinical course.

## 2. Materials and Methods

### 2.1. Gene Selection

Our Return of Results Committee (RORC) and clinical domain management teams compiled a list of genes that would be managed through our population health architecture (Appendix A). This list included the ACMG secondary findings [11], Clinical Pharmacogenetics Implementation Consortium (CPIC) Level 1 and 2 pharmacogenes [12] (Table A2), and other domain-specific genes that were determined to be actionable through our RORC. These were divided into tiers (Table A1). Tier 1 genes consist of those with the strongest clinical evidence and include the Centers for Disease Control and Prevention (CDC) tier one conditions [13] and hereditary hemochromatosis (HH) [14]. Tier 2 genes were divided into two sub-tiers. Tier 2a includes those genes that were considered to have strong levels of evidence for clinical actionability and that had medical management guidelines available. This tier consists of all genes from ACMG 3.0 secondary findings list. Tier 2b genes were those supported by moderate evidence where medical management was available that had not been included in ACMG recommendations. Tier 3 consists of genes with preliminary evidence available for clinical actionability. Results in these genes are to be assessed in the clinical context of the patient and determined for return on a case-by-case basis. Genes selected by the RORC were reflective of the collective clinical expertise of the committee with anticipation that the list would expand as specialists from new clinical domains were added to the committee.

### 2.2. HerediGene Sequencing Program

Whole genome sequencing was performed on 26,302 patients enrolled in the HerediGene Program. These sequences were analyzed for likely pathogenic variants in the actionable genes outlined in Table A1. Of these reportable findings, 5 diseases were targeted for the initial implementation of the population health infrastructure. We developed our framework with the implementation of HH return of results and then scaled our processes to both cardiology and oncology domains simultaneously. Within cardiology we returned pathogenic variants in Familial Hypercholesterolemia (FH), and Hypertrophic Cardiomyopathy (HCOM). Lynch Syndrome (LS) and *BRCA1* and *BRCA2* Hereditary Breast and Ovarian Cancer (HBOC) were selected as the first conditions to return in the oncology domain.

### 2.3. Clinical Validation of Research Data

As the whole genome sequencing in the HerediGene research program does not take place in a CLIA-certified laboratory, all findings were confirmed in a CLIA-certified laboratory before being used for clinical care. Patients from the study were re-contacted through letters explaining the potential risk of their research finding and provided contact information for one of our study genetic counselors to discuss confirming their result. Follow-up phone calls were made if there was no response to the letter. Discussions about risk were very broad at this point in the pathway, given the uncertainty of the unconfirmed result. After patients were contacted and agreed to have their findings confirmed, buccal swab kits were sent to them to submit DNA samples back to the laboratory to confirm their findings.

### 2.4. Population Health Framework

**Figure 1:**
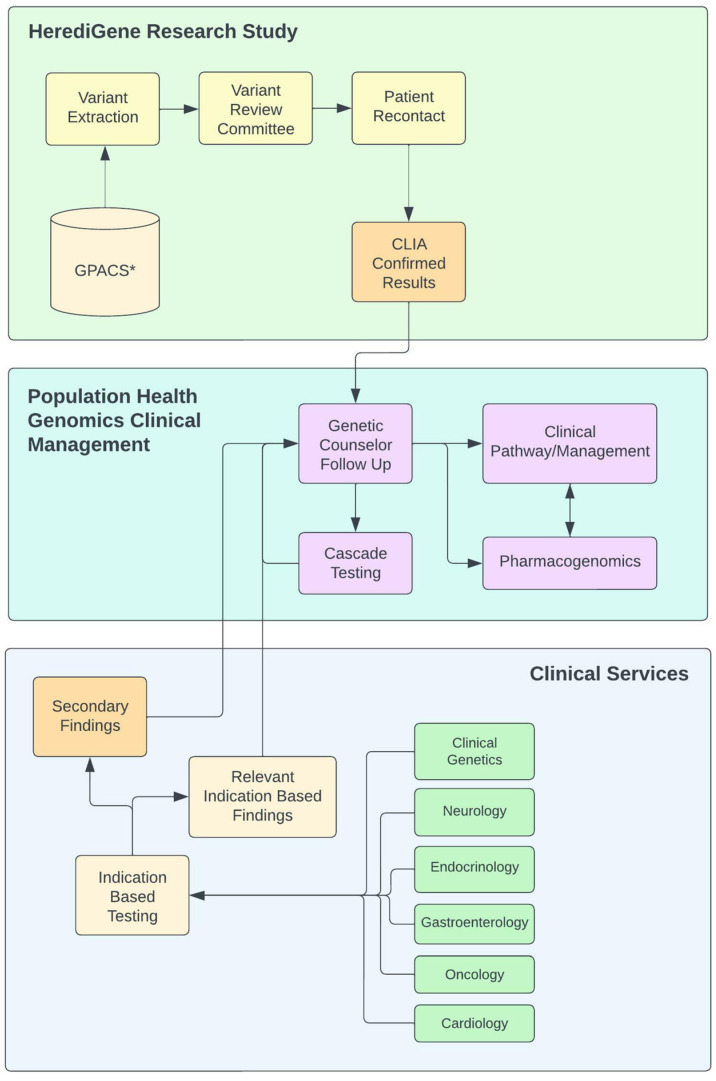
Diagram of population health genomics clinical workflow. * (GPACS) Genotype-Phenotype Archiving and Communication System.

Through the RORC, an overarching architecture was designed to manage clinically actionable findings from genomic testing. This framework was designed to manage findings from multiple sources allowing for the flow of genomic results from both clinical and research pathways. A basic flow diagram is shown in Figure 1. Actionable findings from the HerediGene study would come into the system after CLIA-certified laboratory confirmation. Both primary and secondary findings from clinical genomic sequencing could be routed into this framework for management. The framework was designed to manage both clinically actionable variants for Mendelian disease and pharmacogenomics variants that might impact the patient’s clinical management, particularly in relation to their clinical disease or disease predisposition. Our RORC developed a checklist for our devised system that must be completed prior to beginning the process of returning each gene/disease. Each element of the checklist represented a critical component of the management process for a gene/disease. Upon the completion of the checklist there is a process in place to manage a patient with variants in the given gene/disease. This provided us with a scalable process. Some elements of the checklist are left as “to be determined,” such as clinical decision support (CDS) as our electronic medical record (EMR) vendor had not yet implemented the use of structured genetic data for CDS. The twelve elements of the checklist are as follows: the clinical domain management team; the primary patient and provider contacts; the variant review committee; variant selection guidelines; patient-facing information; provider facing information; pharmacogenomics implications; defined clinical care pathways; clinical decision support models; outcomes definition and measurement; genetic counseling note template; and informational video location. Each of these checklist elements are described more fully in the sections below.

### 2.5. Clinical Domain Management Team

This team determines the genes to be returned and provides guidance in prioritization and clinical management of each condition. They also provide oversight for the creation of patient- and physician-facing information and any clinical decision support. This team is assembled for each clinical domain (i.e., oncology, cardiology, metabolics, endocrinology, etc.). One of their critical functions is to lay out clinical pathways for the management of each disease or genetic predisposition. This team consists at a minimum of a clinical/molecular geneticist, a genetic counselor, and a clinical specialist in the domain of interest. This team is led from an operational standpoint by a genetic counselor. A specialist physician provides the final approval on all materials and clinical management guidelines. There may be considerable overlap of personnel between teams especially for conditions in the same clinical domain.

### 2.6. Primary Patient and Provider Contacts

Primary contacts were designated to ensure patients and providers had someone to reach out to for questions regarding a given condition. This includes a phone number and an email address where support can be obtained. These may or may not be the same for patients versus clinical providers.

### 2.7. Variant Review Committee (VRC)

Our variant review committee is gene/disease specific and consists at a minimum of a bioinformaticist, a variant scientist, a clinical/molecular geneticist, a genetic counselor, and a clinical specialist in the disease of interest. This review committee is responsible for defining parameters to select variants for the return of results bioinformatic pipeline and to curate variants that fall below the ClinVar 3-star classification, and meet automated criteria as outlined in the ‘Variant Selection Guidelines’ section. This review may consist of clinical correlation of variants through the review of the medical records of subjects that harbor the variant.

### 2.8. Variant Selection Guidelines

These are defined guidelines that determine the genes to assess for a given condition, any specific domains/exons to assess, and any specific bioinformatics methods for variant calling. Where consensus statements exist, they are utilized. This information is not static and is monitored as more information becomes available. Our default bioinformatics pipeline was designed to capture variants from genes that were designated in our return of results process into three different tiers. The first tier consists of those variants with ClinVar three-star classification. These are automatically determined to be pathogenic without further human review and are sent into our return of results pipeline automatically. The second tier consists of variants with two-star classification of pathogenic or likely pathogenic. This tier requires manual review through the designated variant review committee. The third bucket consists of variants that had less than a two-star classification or were not classified in ClinVar that were determined by in silico prediction models to be pathogenic. We set up individual bioinformatics classifications for each gene for this tier when there was gene-specific variant calling criteria where in silico predictors have insufficient predictive value. For example, the presence of a specific exonic region of functional consequence or occurrence of a gain of function variant. The last tier bioinformatics pipeline is designed through our variant review committee using both bioinformatics and clinical expertise. All variants from this last tier are reviewed by the variant review committee and may be clinically correlated with patient data as part of that process. For example, a patient with a variant in a gene causing FH that is not classified in ClinVar and has moderate impact scores from in silico prediction tools may be returned if the patient has high cholesterol, a strong family history of heart disease, and no other known genetic variants that explain the condition. All oncology variants with a ClinVar rating of less than 3 stars receive an additional review from an outside laboratory prior to confirmation due to the invasive nature of the interventions that may accompany a diagnosis. In addition, for all cancer variants there is a shared database maintained between IH and The University of Utah’s Huntsman Cancer Institute that contains every variant that has been seen clinically between the two healthcare systems and the classification that it was assigned. These two healthcare systems together provide care for the majority of the Utah patient population.

### 2.9. Standardized Patient- and Provider-Facing Information

Separate standardized information sheets were created for patients and healthcare providers. These sheets were required to be easily interpretable by non-genomics experts. Patient information sheets were designed to provide patients with a relevant summary of their disease predisposition and its management. Patient focus groups were conducted by an outside commercial entity that designed the initial sheets to ensure that they catered to patient needs and gave them most of the pertinent information in a condensed format. Primary care physicians (PCPs) are critical to the management of the patient’s condition and ensuring they follow through on the patient’s preventative care. In some cases, the PCPs are the physicians that will deliver the preventative measures until the patient needs to see a specialist. It was critical not only to organize specialty clinics for the management of patients, but also to provide information to PCPs to manage the conditions in cases where the patient did not have access to clinics or opted to have their PCP manage the condition. Because PCPs must ingest the information in a short amount of time and see the patient in a very truncated visit, it was critical to design the information sheets in a condensed format that could be acted on quickly with the ability to reference further information where needed.

### 2.10. Pharmacogenomics Implications

Pharmacogenomics results are planned to be returned in cases where there is a clinical indication and associated medication use that involves a pharmacogenomics variant that may impact clinical care. Our first implementation of pharmacogenomics will involve statin prescribing for patients with familial hypercholesterolemia. Pharmacogenomics implications are reviewed by Intermountain Healthcare’s pharmacogenomics pharmacy and therapeutics subcommittee and are CLIA-laboratory confirmed through the Intermountain Precision Genomics commercial RxMatch^®^ testing platform [15].

### 2.11. Defined Clinical Care Pathways

**Figure 2:**
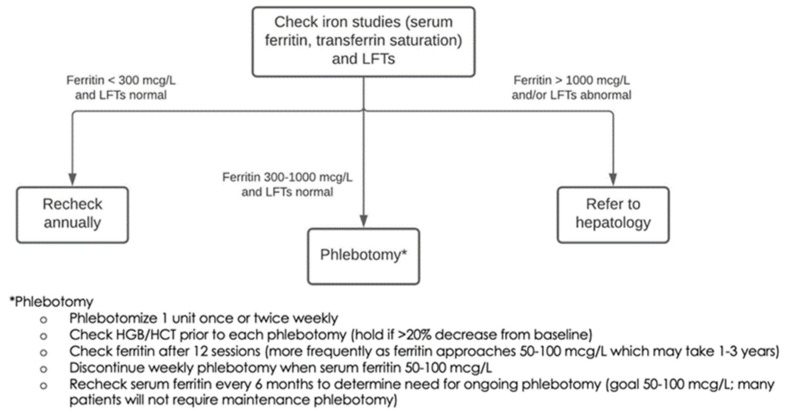
Diagram from our provider-facing information given to primary care providers for management of hereditary hemochromatosis.

Clinical care pathways consist of a visit with a genetic counselor for counseling and cascade testing followed by designated clinical pathways for long-term clinical management and prevention of the disease manifestations associated with the genetic condition. These pathways are designed by the clinical domain management team, sometimes with the help of other specialists. In some cases, such as with HH, management could be initially performed through a PCP and only routed to specialty clinics once the condition, in this case ferritin levels, reaches a critical level (see Figure 2). PCPs are provided with educational materials that outline clear and concise management guidelines. Genetic counselors trained in related specialties are involved where available. In cases where there were no specialists available (GI, endocrine) genetic counselors were trained on conditions in collaboration with a specialist physician. All clinical encounters in this framework are delivered through telemedicine, except for the latter points in clinical pathways where a patient develops symptoms/disease and is required to see a physician for treatment. A robust clinical telemedicine program was developed across the Intermountain Healthcare System at the onset of COVID-19 and this program was leveraged to launch our population health genomics program.

### 2.12. Clinical Decision Support (CDS)

CDS is critical for the long-term scaling of these processes. Without automation, the requirements for managing clinical care of all conditions discovered can be overwhelming. CDS support may include scheduling reminders for preventative maintenance tasks or active CDS for prescribing as completed in primary author’s prior work [16]. For the current implementation this is a placeholder as we work with our EMR vendor to enable this capability.

### 2.13. Outcomes Definition and Measurement

An outcome tracking system was developed to measure effectiveness of preventative measures and to ensure that patients were following up appropriately and receiving necessary care. For each gene/disease, variables and tracking intervals are defined prior to implementing return of results. Tracking is performed in our custom population health database.

### 2.14. Genetic Counseling Note Template

Genetic counseling note templates are set up to ensure consistent delivery and documentation of information and to expedite the process of documenting the patient encounter. This template can be accessed by any genetic counselor in the healthcare system.

### 2.15. Informational Videos

To maximize the efficiency of genetic counseling visits, animated videos were developed to explain many of the frequent topics. The patient could watch these videos during or before the encounter. In addition to saving genetic counselor time, this ensured that patients received consistent and complete information about the given conditions, with the opportunity to ask questions of the genetic counselor after watching the videos.

## 3. Results

### 3.1. Initial Implementation

We identified actionable variants in 1419 (5.4%) patients in the sequenced population. We completed return of results for all HH, HFE pCys282Tyr homozygous patients identified in our initial cohort (*n* = 164). After developing an architecture for HFE and learning from our first implementation, we launched oncology and cardiology pipelines in parallel. We utilized different clinical teams but maintained the same informatics team and clinical geneticist for both projects. These parallel projects introduced unique variant interpretation and classification pathways to our pipeline. We developed complete processes for each pipeline and began the return of results process for patients with variants that predisposed to disease in these areas. Clinical domain management teams and variant review committees were formed for the domains of gastroenterology, oncology, cardiology, endocrinology, and metabolic genetics. Through these teams we produced management guidelines and clinical pathways for more than 58 genes and their associated genetic conditions at the time of submission. Our variant review committee proved particularly useful in determining variants to return for the TTN gene, a new gene in the ACMG list whose variant interpretation guidelines still have some ambiguity. Our architecture allowed patients from any part of the clinical system to have their secondary findings plugged into and managed through well-established clinical pathways that did not require a medical geneticist.

We developed specialty clinics for the management of many of the conditions, particularly oncology, cardiology, and gastroenterology. Clear-cut clinical guidelines were developed to allow for primary care management for some conditions until specialty care was necessary. These specialty clinics are run primarily by advanced practice providers who follow patients for long-term management of their genetic findings. Advanced practitioners were used in cardiology and oncology to run specialty clinics with referral to physicians in cases where indicated.

Cascade testing is managed by genetic counselors that return the results to families. This process is highly dependent on individuals notifying other family members, so the focus is to provide the individual with all the pertinent information they need to give to their at-risk relatives. Family letter templates that include information about the condition of interest, the recommendation for cascade genetic testing, and information on how to contact our team and receive testing and services have been created for many of the conditions.

### 3.2. Scaling the Framework

After completing our first batch of HFE variants, we scaled the process to two other clinical domains (cardiology and oncology) independently and simultaneously. Our framework translated well into the other clinical domains and the scaling was fairly seamless once the checklist was complete. The major difference was the involvement and necessity of the VRC. The VRC for HH only needed to determine early on that only individuals homozygous for the common HFE C282Y variant would be returned as it was rare for other genotypes and compound heterozygotes to develop disease. All variants outside of 3-star ClinVar variants for cardiac and oncological conditions require committee review. Oncological variants were subjected to an independent review from an outside laboratory prior to patient recontact and confirmation. Given the invasive nature of some of the preventative measures for cancer predisposition syndromes, extra caution was taken to ensure variants that were returned were of high specificity. Of note, it was decided through our VRC to study HFE compound heterozygotes to determine if we could detect when they might need management as complications are rare but do occur [14]. Through our return of results program, we did find one compound heterozygote with very high iron levels (1956 mcg/dL) and cirrhosis.

### 3.3. Clinical Integration

Reception and integration of clinical programs varied by department. The GI department faced little clinical impact from the program as most patients with HFE findings were managed through their PCPs until they reached ferritin levels high enough for referral. They did not have a genetic counseling resource and were happy to engage in our processes as it provided access to previously unavailable services to their department. The cardiovascular team had well-established genetics protocols and management pathways with many specialty preventative clinics. Rather than replace these existing clinics, we integrated them into our overarching architecture. There was some reorganization and modifications made to materials and guidelines to standardize pathways to our overall clinical approach. This included the redevelopment of patient and provider-facing materials. In contrast, the oncology team, who had a large team of genetic counselors to assist with return of actionable oncological findings, had a clinical team whose focus was on oncological treatment rather than prevention. This department fully embraced our new approach and helped develop nurse practitioner pathways for patient management. This freed up clinical personnel to focus on treatment while developing a framework and pathways to manage healthy patients and the prevention of disease.

### 3.4. Patient Reaction

At the time of submission 479 results had been initiated through our pathway, out of these, 90 (19%) had a known diagnosis related to their genetic finding on chart review. 29 out of the 479 (6%) of the participants had passed away since enrollment. 174 participants had responded to our recontact attempts at the time of submission. Patients overall expressed interest in and positive views of the program. There were 31 (6.5%) participants who declined follow-up confirmation because they were already aware of their genetic condition and felt they were being managed adequately. Alternatively, with HH and FH, there were 12 patients who had a clinical diagnosis of the condition but who had not to their knowledge had genetic testing and were interested in clinical confirmation testing for that reason. The majority of participants that have responded to our recontact attempts (114/174; 66%) were not aware but were interested in confirming their results. One individual opted out of receiving them due to anxiety, eight declined to follow up because the “timing was bad”, and eight gave no reason for declining their results.

### 3.5. Pediatric Considerations

The HerediGene Children’s Study (HCS) was launched approximately 18 months following the overall HerediGene population study. HCS, which included children and infants from birth through 17 years of age, required more personnel resourcing for the consent processes, given the additional complexities regarding informed consent and assent in minors. Another resource developed for the HCS was the use of buccal cheek swabs as a sample source to avoid blood draws. HCS was initially offered only at the children’s hospital (Primary Children’s Hospital), in order to ensure the availability of research-trained phlebotomists, laboratory personnel trained in pediatric specimen handling, and to provide a higher number of potential enrollees. Following optimization of workflows, HCS was offered at other high-volume pediatric sites, including maternity delivery wards and neonatal intensive care units.

Recognizing the additional complexities for workflow, result interpretation, and return of results, of HCS, HerediGene partnered with the physicians and staff (genetic counselors, laboratory staff, etc.) at the children’s hospital. A separate HCS team met weekly to discuss operations; the HCS team took part in the overall HerediGene committee and RORC; and a separate HCS Ethics and Community Oversight Board was created and met on a regular basis.

HCS return of results followed the same initial pathway as for HerediGene. However, concurrently with the identification of a pathogenic variant in a child, the appropriate pediatric specialty team (e.g., pediatric cardiology, pediatric neurology, etc.) was informed of the result. At the time the child and family were informed of the result a referral was offered to the pediatric specialist and to a genetic counselor trained in the pediatric specialty area.

### 3.6. Informatics Framework

Informatics was critical for the implementation of our architecture, including genomic data repositories, bioinformatics pipelines, databases, and custom clinical applications for management of the return of results program and the clinical management of patients detected through the program. Through these processes we identified the need for technical architectures in seven key areas to enable the scaling of population genomics: 1. Storage and retrieval of patient genomic sequence data. 2. Clinical variant assessment/interpretation/monitoring pipelines. 3. Active and passive genomics-specific clinical decision support. 4. Patient communications tools to facilitate return of results and subsequent management. 5. Management of clinical workflows related to variants returned. 6. Outcomes tracking systems 7. Tools for facilitating and managing cascade testing.

## 4. Discussion

We describe the development and implementation of a system for the use of genomic information in clinical implementation for population health. Our preliminary results are promising and show actionable pathogenic variants for selected genes (Table A1) in nearly 5.4% of the patients in our sequenced population. This percentage demonstrates not only the magnitude of the impact genomics can have on patient health but also demonstrates the need for an architecture that scales to manage genomic data for a large number of patients.

The development of the population genomics framework allowed us to directly link our patients who had secondary findings from indication-based testing, directly into clinical management pathways. This is critical to the scaling of the use of whole genome or exome sequencing throughout the system as secondary findings are a major challenge and workload for clinical genetics and the inability to handle these findings can discourage widespread adoption of sequencing. Of note, we had completed our first gene list prior to the release of ACMG 3.0 recommendations [11]. All of the genes included in the new recommendations were already on our list with the exception of *RPE65*, a retinal dystrophy gene with a new gene therapy. We anticipate that with several gene therapies in the pipeline, other genes will rapidly be identified for inclusion. We are now monitoring these pipelines closely both for future actionability and for the ability to enroll our patients in clinical trials.

The first two identified deficiencies include (1) Storage and retrieval of patient genomic sequence data, and (2) Clinical variant assessment/interpretation/monitoring encompass domains that fall somewhere in between the laboratory and clinic. Most genetic data reside in the laboratory where it is assessed for a particular indication and where secondary findings are returned only while returning the primary indication for which the test was ordered. However, to facilitate the continued use of genomic data for clinical purposes, there needs to be accessible storage within the clinical domain that can be continually assessed for variants that can impact the patient’s care as more knowledge becomes available (e.g., reclassification of variants of unknown significance or newly described gene-disease relationships). We have developed such a system, but the current utilization of the system requires a collaboration between the clinical team and the laboratory team for interpretation and monitoring of variants for clinical use. This was possible because the sequencing laboratory is part of Intermountain Healthcare. To further scale this outside of our healthcare system we would need to develop models that would operate with the laboratory and clinic as completely separate entities.

Our current EMR vendor does not have native support for structured genomic data so the process of creating clinical decision support on such data was very complicated. We developed a prototype of both active and passive decision support that work through the import of genomics variants as discrete custom lab values. We consider this a work-around while we engage with our EMR vendor to encourage the adoption of standards-based genomic data structures.

Communication with patients to return their results is very time consuming and, considering the percentage of the patient population that is impacted, automating parts of this process will be necessary to scale this process. We assessed several existing platforms to optimize communication, and ultimately developed a custom prototype chatbot based on the Microsoft Health Bot platform [17] to help facilitate these clinical workflows. Several other platforms were in use at Intermountain, but none fully met our needs.

We developed clinical workflows for each of the variants returned and developed our own internal database to manage patients through these workflows. We also developed specialized clinics to facilitate the treatment and management of these patients. We are working on integration of our custom-built tool into the EMR to streamline our processes.

Monitoring outcomes is important for public health genomics to ensure that the care being delivered is having a significant impact. We did not have any tools that accurately captured this information based on genomic variants, so we built our own custom database to monitor patient outcomes and automatically capture data from the EMR.

Cascade testing is an important part of population health genomics that involves the testing of additional family members who may harbor the familial variant that increases disease risk. These family members could be a part of the same healthcare system or may even be in another state or country. HIPAA regulations require that the proband initiate any outreach to connect with other family members to see if they would like to receive testing. Systems that help facilitate this process are critical to the impact of genomics beyond the current healthcare system.

While the implementation described is primarily for germline variation, our current architecture is designed to track and manage somatic variants as well. IH has a robust system for the clinical management of somatic variation that has been previously described [18]. We plan to more fully integrate these processes with an underlying technical architecture as our platform evolves.

## 5. Conclusions

Implementation of genomics at the scale of population health utilizes complicated technologies and processes that for many health systems are not supported by current information systems or in existing clinical workflows. To scale and provide population health utilizing genomic information requires a substantial clinical framework that is backed by informatics tools to facilitate the flow and management of data. Our work represents an early model that has been successful in scaling to 29 different genes and four clinical domains. More work needs to be done to optimize informatics tools; and to identify best practices for translation to smaller healthcare systems.

## Data Availability

Not applicable.

## Appendix A

Actionable mendelian disease risk genes and pharmacogenes chosen by committee for deployment through the population health genomics architecture.

**Table A1:** Genes selected for return to patients by our return of results committee divided by tier.

**Tier 1**		
	Familial hypercholesterolemia	*APOB*
		*LDLR*
		*PCSK9*
	Hereditary breast and ovarian cancer	*BRCA1*
		*BRCA2*
	Hereditary hemochromatosis	*HFE*
	Lynch Syndrome	*MLH1*
		*MSH2*
		*MSH6*
		*PMS2*
**Tier 2A**		
	Aortopathies	*ACTA2*
		*FBN1*
		*MYH11*
		*SMAD3*
		*TGFBR1*
		*TGFBR2*
	Arrhythmogenic right ventricular cardiomyopathy	*DSC2*
		*DSG2*
		*DSP*
		*PKP2*
		*TMEM43*
	Biotinidase deficiency	*BTD*
	Cardiomyopathies	*ACTC1*
		*FLNC*
		*LMNA*
		*MYBPC3*
		*MYH7*
		*MYL2*
		*MYL3*
		*PRKAG2*
		*TNNI3*
		*TPM1*
		*TTN*
	Ehlers-Danlos syndrome, vascular type	*COL3A1*
	Fabry’s disease	*GLA*
	Familial medullary thyroid cancer	*RET*
	Hereditary breast and ovarian cancer	*PALB2*
	Inherited cardiac Arrhythmias	*CASQ2*
		*KCNH2*
		*KCNQ1*
		*RYR2*
		*SCN5A*
		*TRDN*
	Hereditary colorectal cancer	*APC*
		*BMPR1A*
		*MUTYH*
		*SMAD4*
	Hereditary hemorrhagic telangiectasia	*ACVRL1*
		*ENG*
	Hereditary paraganglioma-pheochromocytoma syndrome	*MAX*
		*SDHAF2*
		*SDHB*
		*SDHC*
		*SDHD*
		*TMEM127*
	Left ventricular noncompaction	*TNNT2*
	Li-Fraumeni syndrome	*TP53*
	Malignant hyperthermia	*CACNA1S*
		*RYR1*
	Maturity-onset of diabetes of the young	*HNF1A*
	Multiple endocrine neoplasia	*MEN1*
	Neurofibromatosis, type 2	*NF2*
	Ornithine carbamoyltransferase deficiency	*OTC*
	Peutz-Jeghers syndrome	*STK11*
	Pompe disease	*GAA*
	PTEN hamartoma tumor syndrome	*PTEN*
	Retinoblastoma	*RB1*
	RPE65-related retinopathy	*RPE65*
	Tuberous sclerosis complex	*TSC1*
		*TSC2*
	Von Hippel-Lindau syndrome	*VHL*
	WT1-related Wilms tumor	*WT1*
	Wilson disease	*ATP7B*
**Tier 2B**		
	Aortopathies	*MYLK*
		*LOX*
		*PRKG1*
		*SMAD2*
		*TGFB2*
		*TGFB3*
	Arrhythmogenic right ventricular cardiomyopathy	*JUP*
		*PKP2*
	*BAP1* tumor predisposition syndrome	*BAP1*
	Birt-Hogg-Dubé syndrome	*FLCN*
	Cardiomyopathies	*ACTN2*
		*BAG3*
		*CSRP3*
		*DES*
		*DMD*
		*LAMP2*
		*PLN*
		*RBM20*
	*DICER1* tumor predisposition	*DICER1*
	Familial hypercholesterolemia	*LDLRAP1*
	Hereditary breast and ovarian cancer	*ATM*
		*BRIP1*
		*CHEK2*
		*RAD51C*
		*RAD51D*
	Hereditary colorectal cancer	*POLE*
	Hereditary diffuse gastric cancer	*CDH1*
	Hereditary skin cancer	*CDKN2A*
	Hereditary transthyretin amyloidosis	*TTR*
	Heritable pulmonary arterial hypertension	*BMPR2*
		*TBX4*
	Hypertrophic cardiomyopathy	*TNNC1*
	Inherited cardiac arrhythmias	*CALM1*
		*CALM2*
		*CALM3*
		*KCNE1*
	Lynch syndrome	*EPCAM*
	Metachromatic leukodystrophy	*ARSA*
	Neurofibromatosis, type 1	*NF1*
	Prostate cancer	*HOXB13*
	Pulmonary venoocclusive disease	*EIF2AK4*
	X-Linked adrenoleukodystrophy	*ABCD1*
Tier 3		
	Aortopathies	*COL5A2*
		*EFEMP2*
		*FBN2*
		*FOXE3*
		*GATA1D*
		*MAT2A*
		*MFAP5*
		*NOTCH1*
		*PLOD1*
		*SKI*
		*SLC2A10*
	Bloom Syndrome	*RECQL3*
	Capillary malformation-ateriovenous malformation	*RASA1*
	Cardiac Arrhythmias	*ANK2*
		*CACNA1C*
		*CACNB2*
		*GPD1L*
		*HCN4*
		*KCNA5*
		*KCNE2*
		*KCNE3*
		*KCNJ2*
		*NKX2-5*
		*SCN1B*
		*SCNB3*
		*SNTA1*
	Cardiomyopathies	*ABCC9*
		*CAV3*
		*CRYAB*
		*DOLK*
		*EYA4*
		*FKRP*
		*HRAS*
		*RAF1*
		*SGCD*
		*TAZ*
		*TCAP*
		*VCL*
	Carney complex	*PRKAR1A*
	Glycogen storage disease	*AGL*
	Hereditary Multiple Osteochondromas	*EXT1*
		*EXT2*
	Hereditary colorectal cancer	*AXIN2*
		*GREM1*
		*MSH3*
		*NTHL1*
		*POLD1*
	Hereditary paraganglioma-pheochromocytoma syndrome	*SDHA*
	Maturity-onset of diabetes of the young	*ABCC8*
		*HNF1B*
		*HNF4A*
		*KCNJ11*
	Nijmegen breakage syndrome	*NBN*
	Nevoid basal cell carcinoma syndrome	*PTCH1*
		*SUFU*
	Parathyroid Cancer	*CDC73*
	Pulmonary Arterial Hypertension	*CAV1*
		*SMAD9*
	Rhabdoid tumor predisposition syndrome	*SMARCA4*
		*SMARCB1*
	Rothmund-Thomson syndrome, type 2	*RECQL4*
	RUNX1 familial platelet disorder with associated myeloid malignancies	*RUNX1*
	Skin Cancer	*CYLD*
		*ERCC1*
		*ERCC2*
		*ERCC3*
		*ERCC4*
		*ERCC5*
		*MITF*
	Systemic Primary Carnitine Deficiency	*SLC22A5*
	Thrombosis	*CBS*
	Werner Syndrome	*WRN*

**Table A2:** CPIC level 1 and level 2 pharmacogenes.

CPIC Pharmacogenes							
*ABCG2*	*BCHE*	*CYP2C19*	*DPYD*	*HLA-B*	*MT-RNR1*	*OTC*	*SLC6A4*
*ABL2*	*CACNA1S*	*CYP2C9*	*G6PD*	*HLA-DRB1*	*MTHFR*	*POLG*	*SLCO1B1*
*ADRB1*	*CFTR*	*CYP2D6*	*GBA*	*HPRT1*	*NAGS*	*RYR1*	*TPMT*
*ASL*	*CPS1*	*CYP3A5*	*GRK5*	*IFNL3*	*NAT2*	*SCN1A*	*UGT1A1*
*ASS1*	*CYP2B6*	*CYP4F2*	*HLA-A*	*IFNL4*	*NUDT15*	*SLC28A3*	*VKORC1*
